# Smart Humidly Adaptive Yarns and Textiles from Twisted and Coiled Viscose Fiber Artificial Muscles

**DOI:** 10.3390/ma15238312

**Published:** 2022-11-23

**Authors:** Mingrui Guo, Yangyang Peng, Zihan Chen, Nan Sheng, Fengxin Sun

**Affiliations:** 1 MOE Key Laboratory of Eco-Textiles, Jiangnan University, Wuxi 214122, China; 2 Laboratory of Soft Fibrous Materials, Jiangnan University, Wuxi 214122, China; 3 College of Fashion Design, Jiaxing Nanhu University, Jiaxing 314001, China

**Keywords:** artificial muscles, smart textiles, torsional actuation, contractile actuation, viscose fibers

## Abstract

The self-adaptive nature of smart textiles to the ambient environment has made them an indispensable part of emerging wearable technologies. However, current advances generally suffer from complex material preparation, uncomfortable fitting feeling, possible toxicity, and high cost in fabrication, which hinder the real-world application of smart materials in textiles. Herein, humidity-response torsional and tensile yarn actuators from twisted and coiled structures are developed using commercially available, cost-effective, and biodegradable viscose fibers based on yarn-spinning and weaving technologies. The twisted yarn shows a reversible torsional stroke of 1400° cm^−1^ in 5 s when stimulated by water fog with a spraying speed of 0.05 g s^−1^; the coiled yarn exhibits a peak tensile stroke of 900% upon enhancing the relative humidity. Further, textile manufacturing allows for the scalable fabrication to create fabric artificial muscles with high-dimensional actuation deformations and human-touch comfort, which can boost the potential applications of the humidly adaptive yarns in smart textile and advanced textile materials.

## 1. Introduction

As the intense heatwave swept across the world in recent years, the extreme temperatures are putting people at increased risk of illness and causing energy shortages [1,2]. Various novel fabrics for personal thermal management (such as radiative cooling materials [3,4], thermochromic fibers [5], high thermal conduction, and convection textiles [6,7]) have been developed for cooling clothes. In particular, moisture-sensing smart textiles that can positively respond to human perspiration and ambient environment, making adaptable changes and thereby increasing the wearer’s thermal-wet comfort, are highly desirable [8,9,10].

Recent advances in fiber-based artificial muscles pave a convenient way to design humidly adaptive textiles for personal moisture and thermal management [11,12]. Various materials, such as carbon nanotubes [13,14], shape memory alloy [15], graphene [16], polymers [17], and their composites [18,19], have been used to fabricate yarn artificial muscles. However, current materials generally suffer from either the high cost and potential chemical toxicity in processing or harsh stimulus conditions and poor wearable comfortability, which hinder their practical application in textiles. More recently, natural fibers including cotton, linen, silk, and wool [20,21,22,23] have been used to fabricate yarn muscles, but the variability of the natural fibers results in unmanageable and unstable actuation behavior. It is desirable to find other environmentally friendly, affordable, and renewable materials to prepare yarn muscles with steady and programable actuation performance.

Regenerated cellulosic fiber (viscose fiber), one of the most widely used fibers in modern textiles, has attracted great attention from chemical fiber and textile industry due to its skin-friendly, biodegradability, and low cost (about USD 1.5 per kg) [24]. Moreover, the excellent hygroscopicity and outstanding volume expansion of viscose fibers upon absorbing water or moisture make them a promising candidate for moisture-sensing yarn artificial muscles [25,26]. However, the real-world application of such yarn muscles to smart textiles is still less studied because of the complexity of the structural manipulation of fabrics. Recent advances in applying commercially available viscose fibers to coiled yarn muscles have served as the foundation of fiber-based artificial muscles [25], but the low actuation and recovery speed and small contraction stroke have limited the application of such yarn muscles. Therefore, instead of harnessing advanced smart materials, processing cost-effective and skin-friendly conventional viscose fibers into high-performance humidity-response materials based on hierarchical structure design will be of great significance to promote the development and real-world application of fiber-based artificial muscles in smart textiles.

Herein, we demonstrate the torsional and tensile actuation of twisted and coiled viscose yarn artificial muscles when driven by water fog/moisture. The yarn muscles show reversible actuation when alternatively exposed to wet and dry conditions. The maximum torsional stroke of twisted yarn muscles is up to 1400° cm^−1^, and the maximum torsional speed is 960 rpm. Moreover, the coiled yarn muscles display excellent contractional stroke of up to 900% when driven by water fog, which is more than 25 times higher than that in the previous study [26]. Moreover, the viscose yarns are scaled up to fabrics by topological weaving structures, showing a promising application perspective for smart textiles and intelligent systems.

## 2. Materials and Methods

### 2.1. Materials and Fabrication of Viscose Muscles

The viscose multifilament (150D/30F) was provided by Jilin Chemical Fiber Co., Ltd., China. To fabricate the yarn artificial muscles, a required number of viscose filaments (typically two filaments) was separated from the viscose multifilament. For the torsional yarn artificial muscles, the viscose filaments were first hung with a gauge length of about 25 cm between the rotary jaw and a small clip. The small clip with a weight of 1.2 g is restricted in rotation but can be freely moveable along the filament’s longitudinal direction. By rotating the rotary jaw, 2000 turns were inserted into the viscose filaments, and then the twisted filaments were folded from the middle to form torsional yarn muscles with a self-balanced, double-helical structure. For contractile yarn muscles, the torsional yarn muscles were wrapped around a steel bar to form a coiled structure, followed by a wetting and heating (90 °C for 30 min) process to set the coiled shape of the yarn.

### 2.2. Characterizations

The morphological structures of the viscose filament and artificial muscles were observed using a scanning electron microscope operated at 5 kV accelerating voltage (SEM; SU 1510, Hitachi High-Technologies Corp., Tokyo, Japan). The mechanical property of the viscose filament was measured by a fiber strength extensometer (XQ-2, Shanghai Xinxian Instrument Co., Ltd., China), where the gauge length was 25 cm, and tensile speed was 15 mm min^−1^. The thermal property of the original and heat-treated viscose filaments was characterized by a thermo gravimetric analyzer (TGA-2, Mettler-Toledo, Switzerland) at a heating rate of 20 °C min^−1^ and temperature changing from 30 to 500 °C.

### 2.3. Measurement of Actuation Performance

The driving process of the yarn muscles was performed in a self-made acrylic chamber box with dimensions of 30 cm × 30 cm × 50 cm. The dehumidifier (KJ200G-B01, Gree Electric Appliances, Qingdao, China) was used to control the relative humidity at below 30% (RH) and at 25 °C for the dry state of the viscose muscles. For the driven process, a humidifier (JSQ-F50B1, Little Bear Appliance Co., Ltd., Shunde, China) with a water fog spraying speed of 0.05 g s^−1^ was used to wet the muscles if not specified. The whole process was captured by a high-speed camera (Micro LAB-110, Ametek Co., San Diego, CA, USA) and analyzed by video processing. The tensile yarn muscles have two modes of tensile stroke, that is, contraction stroke (*S_c_*) and extension stroke (*S_e_*), which are calculated as

(1) $$S_{c}=\frac{-\Delta L}{L_{0}}$$

(2) $$S_{e}=\frac{\Delta L}{L_{0}}\;$$

where $\Delta L=L-L_{0}$, is the length change in the axial direction of the coil yarn muscles, and *L*_0_ is the original length of the yarn muscles.

## 3. Results and Discussion

### 3.1. Structures and Properties of Viscose Filaments

**Figure 1:**
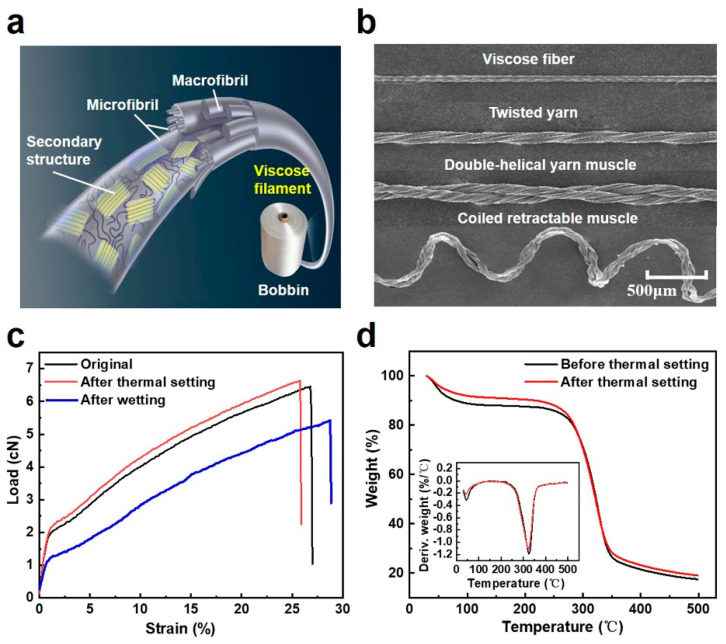
The structure and performance of viscose filaments. (**a**) Hierarchical structure of a viscose filament. (**b**) The SEM image of viscose filaments in different morphological states. (**c**) Typical tensile curves of a non-twist single viscose filament with different treatment states. (**d**) TGA curves of viscose filaments before and after thermal setting.

Previous studies have revealed that the actuation mechanism of yarn artificial muscles originates from the reversible volume expansion, especially anisotropic expansion, of twisted filaments [17,27]. Regenerated cellulose fibers, mainly including lyocell, model, and viscose, show interesting hierarchical architecture with secondary structures, microfibril, and microfibril [28], as shown in Figure 1a, which enables the imposition of anisotropic alignment within the filaments, thereby enhancing anisotropic dimensional changes during the stimulation process. The cellulose polymer generally has high moisture-absorption property due to abundant hydroxyl groups in the anhydrous glucose unit, and the water absorption causes outstanding swelling [29]. These merits make regenerated cellulose filaments an ideal candidate for fabricating moisture-sensing actuators. Instead of using lyocell and modal, we here selected the viscose that is spun by a conventional wet-spinning process from cellulose pulp (degree of polymerization: 450–550) to fabricate twisted yarn muscles because a viscose filament generally has a gradient structure along the radial direction with a decreasing degree of crystallinity but an increasing degree of orientation from filament center to filament surface because of the extrusion features of the wetting-spinning process [30], which collectively contribute to the rapid water absorption and outstanding hydroexpansivity of the viscose filaments [31], which is suitable for fabricating the moisture-sensing yarn artificial muscles. Moreover, different from natural fibers, viscose filaments show uniform diameters and can be twisted, folded, and coiled into steady morphologies for torsional and tensile yarn muscles (Figure 1b). Furthermore, the viscose filaments exhibit robust mechanical properties when exposed to a thermal setting under 90 °C for 30 min, as indicated by the similar tensile curves between original and thermal-setting viscose filaments (Figure 1c). Comparisons of TGA curves also confirm that the thermal setting process has little effect on the microstructure of viscose filaments (Figure 1d), whereas the wetted viscose filaments display a lower Young’s modulus and larger elongation compared with that in a dry state. This may be caused by the fact that water molecules infiltrate into the amorphous region of cellulose and disrupt the hydrogen bonds, which is also considered the main contribution to the wet expansion of the filaments [25,32].

### 3.2. Torsional Yarn Artificial Muscles

**Figure 2:**
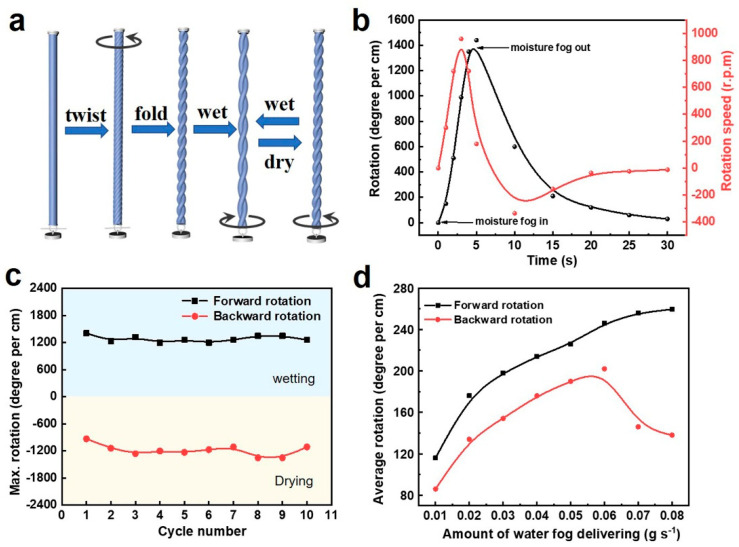
Fabrication and actuation performance of the torsional yarn muscles. (**a**) Illustration of the fabrication of self-balanced torsional yarn muscles. (**b**) Rotation degree (black curve) and rotation speed (red curve) as a function of time upon wetting and drying. (**c**) The durability of the reversible torsion when alternatively exposed to wet and dry over 10 cycles. (**d**) The evolution of average rotation and reverse degree with different amount of water fog delivering.

The wet expansion of viscose filaments can be amplified by inserting high twists into the filaments, and then, a self-balanced double-helical structure can be constructed by folding the twisted viscose filaments in the middle to ply two parts of the filaments via the untwist of initially twisted filaments, thereby forming torque-balanced, moisture-sensing yarn muscles (Figure 2a). Upon wetting, each of the individual twisted filament parts untwists due to the expansion of the filament volume, which generates a torque and causes an increase in the twist of the plied yarn. During the water desorption process, the increased twist in the plied yarn will be released to achieve torsion balance along with an increase of twist in individual filament parts, and thus, the plied yarn returns to its initial balanced state. Therefore, the yarn muscles exhibit reversible torsional actuation performance when alternatively exposed to wet and dry environments. In the torsional actuation, a triangle paddle (~4.8 mg) is tied to the end of the plied yarn muscle for observation purposes, which also provides an isobaric load for the yarn muscle. As shown in Figure 2b, the plied yarn muscle generates a rotation of around 1400 degree per cm in 5 s when stimulated by water fog, showing a maximum rotation speed of 960 rpm, which is comparable to natural fiber muscles, such as cotton fiber (720 rpm) [33], wool fiber (60 rpm) [22], silk (1125 rpm) [19], and certain advanced carbon-based fibers [34,35]. The reversible rotation actuation performance is also observed, and the yarn muscle rotates back to the original state in 25 s upon drying (Figure 2b). Such yarn muscle shows stable cyclic actuation performance over 10 actuation cycles, and the maximum rotation has no significant decrease with the coefficient of variation less than 0.055 for forward rotation and below 0.010 for backward rotation (Figure 2c). Moreover, the torsional stroke of the plied yarn muscles is highly related to the amount of water fog delivering. Enhancing the amount of water fog delivering can increase the rotation degree of the yarn muscles; meanwhile, increased amounts of water fog will condense on the surface of the yarns, which instead decreases the speed of water desorption from the yarn muscles in the drying process, thereby resulting in a slow backward rotation speed upon drying (Figure 2d). Therefore, an optimal amount of water fog delivering is experimentally determined as 0.05 g s^−1^ in this study. Based on this relationship between the rotation and applied water fog, we can also tune the rotation degree and rotation speed of the yarn muscles by controlling the amount of water fog delivering in specific applications.

### 3.3. Tensile Yarn Artificial Muscles

**Figure 3:**
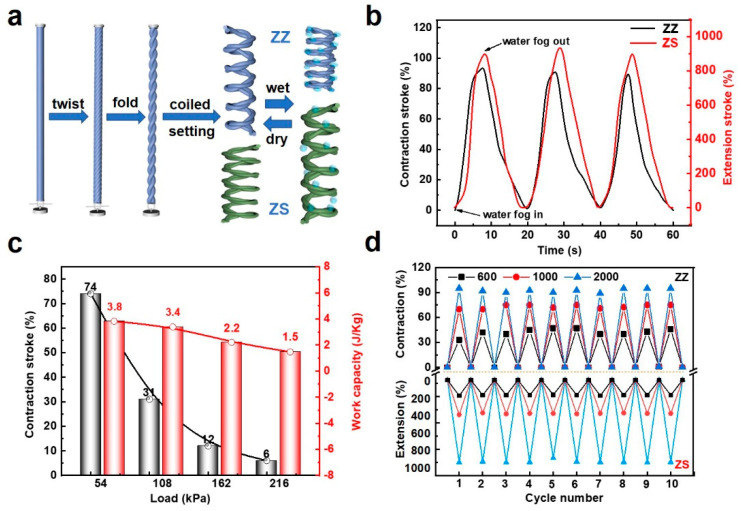
Fabrication and tensile performance of the tensile yarn muscles. (**a**) Illustration of the fabrication process of coiled yarn muscles. (**b**) Tensile stroke as a function of time when alternatively exposed to wet and dry in three cycles. (**c**) Dependence of tensile stroke (black column) on the applied load and the corresponding work capacity (red column). (**d**) Reversible performance over 10 cycles of the tensile yarn muscles with different inserted twists per 25 cm.

The twisted yarns can not only achieve torsional actuation but can also be fabricated into tensile yarn muscles. Following the same routine as the twisted yarn muscles, the viscose filaments are twisted and folded to form double-helical structures. Then, the twisted yarn muscles are further transformed to tensile yarn muscles by wrapping them on a mandrel to form a coiled architecture, as shown in Figure 3a. It should be noted that the chirality of the twisting and coiling determines whether the coiled yarn muscles extend or contract when exposed to water fog. For example, coiling the Z-twisted double-helical yarn muscle on a mandrel in the Z direction forms a homochiral coiled yarn muscle (ZZ muscle), and this yarn muscle is expected to show a contraction deformation upon wetting. This can be due to the expansion of viscose fibers upon wetting, which induces the untwisting of single fibers, increasing the Z-directional twists of the double-helical yarn, and thereby enhancing the Z-directional coiling density to result in a contraction deformation of the coiled yarn muscles. On the contrary, the increase of the Z-directional twists decreases the S-directional coiling density, and thus, heterochiral yarn muscles (ZS muscle) will extend upon wetting. The ZZ and ZS muscles, respectively, show reversible contraction and extension stroke when alternatively exposed to water fog and dry ambient. The maximum contraction stroke of the ZZ muscle is up to 95% for the free-of-load state, while the peak extension stroke of the ZS muscle reaches around 900% when exposed to water fog (Figure 3b). The load applied to the free end of the coiled yarn muscles is an important factor that influences their contraction stroke and work capacity. With the increase of the applied load from 54 to 216 kPa, the contraction stroke of ZZ muscles monotonically decreases from 74 to 6%, while the work capacity varies from 3.8 to 1.5 J kg^−1^ (Figure 3c). Thus, the maximum stroke and work capacity can be achieved at a free-of-load state. Moreover, by increasing the inserted twists, the muscles show an increased stroke (Figure 3d). For convenience, the cyclic experiment is conducted for the ZZ and ZS muscles under the free-of-load state. Both of the ZZ and ZS muscles exhibit high reversibility in contraction and extension strokes (Figure 3d).

### 3.4. Applications

**Figure 4:**
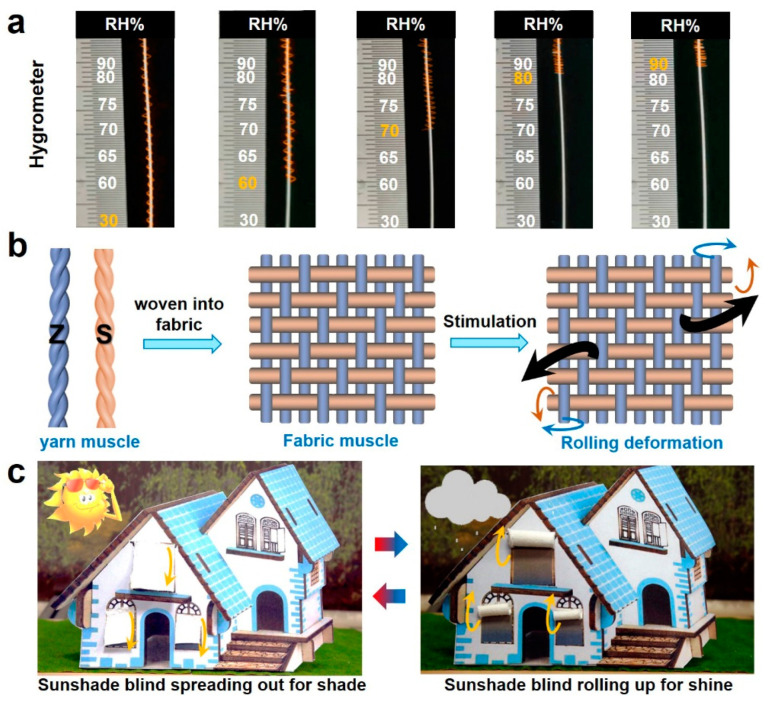
Potential applications and scalable fabrication of fiber-based artificial muscles. (**a**) Natural hygrometer made of coiled yarn muscles. (**b**) Scalable fabrication of twisted yarn muscles into fabric muscles. (**c**) An example of fabric muscle’s application in smart sunshade blind.

The reversible torsional and tensile yarn muscles hold promise as moisture-sensing materials for real-world applications. Figure 4a shows a yarn hygrometer that can promptly respond to different humidity levels with specific contraction strokes to indicate the relative humidity in the ambient. The yarn hygrometer is composed of a coiled yarn muscle as an indicator and a calibrated scale. The coiled yarn muscles are fabricated based on the process of Figure 3a by the ZZ twisting method using viscose filaments and having a twist of 80 turns per cm. With the increase of ambient relative humidity, the ZZ-twisted yarn muscle contracts to indicate the corresponding large value of the relative humidity by its bottom end. When the relative humidity decreases, the yarn muscle extends to show the low humidity value. Such a yarn hygrometer is promising for real-world applications to indicate the real-time relative humidity of the ambient environment without any complicated sensing system and energy supply.

Fabric muscles open new horizons for smart textiles. The twisted torsional yarn muscles can be scaled up to two-dimensional fabrics based on weaving technology. We here select the Z-twisted torsional yarn muscles and S-twisted torsional yarn muscles with 33 tex and 720 twists per meter as warp and weft, respectively, to weave a twill fabric muscle (Figure 4b). The warp density and weft density of the twill fabric muscle woven in this study are 280 yarn counts per 10 cm and 240 yarn counts per 10 cm, respectively. Upon wetting, the torsional yarn muscles of different twisting directions, i.e., Z twist and S twist, within the twill fabric muscle tend to rotate in opposite directions, resulting in unbalanced out-of-plane stress, thereby inducing the bending deformation of the fabric, as shown in Figure 4b, whereas the yarn muscles release the torsion and tend to recover the original balanced state upon drying, and the fabric muscle thereby releases the internal stress to spread out. We here demonstrate that this moisture-driven reversible fabric muscle can be used as a smart sunshade blind, as shown in Figure 4c. The fabric muscle stays in a spread-out state on a sunny day to shield the window from the burning sun, thereby keeping it cool inside of the house. In case of dark light on a rainy day, the fabric muscle will roll up to let more light in (Figure 4c). Therefore, the proposed fabric muscle can be a promising candidate for a variety of applications in intelligent systems and smart textiles.

## 4. Conclusions

The torsional and tensile yarn artificial muscles are fabricated based on twisting and coiling processes using easily available viscose fibers without any further chemical treatment. Reversible and excellent actuation performance of the yarn muscles is demonstrated. The torsional yarn muscles show a maximum torsional stroke of 1400° cm^−1^ and a maximum rotation speed of 960 rpm, which is comparable to reported natural fiber muscles (60–1125 rpm). The coiled yarn muscles exhibit a maximum extension stroke of 900% for ZS-twisted yarn and a maximum contraction stroke of 90% for ZZ-twisted yarn. The yarn hygrometer is also demonstrated using the moisture-sensing coiled yarn muscles, and fabric muscles are further fabricated using torsional yarn muscles to achieve high-dimensional bending deformations when alternatively exposed to wet and dry environments, which promotes the fiber-based artificial muscles for wider applications, such as smart textiles and intelligent systems.

## Data Availability

Not applicable.
